# Prefrontal Ischemia in the Rat Leads to Secondary Damage and Inflammation in Remote Gray and White Matter Regions

**DOI:** 10.3389/fnins.2016.00081

**Published:** 2016-03-02

**Authors:** Nina Weishaupt, Angela Zhang, Robert A. Deziel, R. Andrew Tasker, Shawn N. Whitehead

**Affiliations:** ^1^Department of Anatomy and Cell Biology, Schulich School of Medicine and Dentistry, University of Western OntarioLondon, ON, Canada; ^2^Department of Biomedical Sciences, University of Prince Edward IslandCharlottetown, PEI, Canada

**Keywords:** thalamus, stroke, white matter inflammation, diaschisis, axonal degeneration, microglia

## Abstract

Secondary damage processes, such as inflammation and oxidative stress, can exacerbate an ischemic lesion and spread to adjacent brain regions. Yet, few studies investigate how regions remote from the infarct could also suffer from degeneration and inflammation in the aftermath of a stroke. To find out to what extent far-remote brain regions are affected after stroke, we used a bilateral endothelin-1-induced prefrontal infarct rat model. Brain regions posterior to the prefrontal cortical infarct were analyzed for ongoing neurodegeneration using FluoroJadeB (FJB) and for neuroinflammation using Iba1 and OX-6 immunohistochemistry 28 days post-stroke. The FJB-positive dorsomedial nucleus of the thalamus (DMN) and retrosplenial area (RSA) of the cortex displayed substantial neuroinflammation. Significant neuronal loss was only observed within the cortex. Significant microglia recruitment and activation in the FJB-positive internal capsule indicates remote white matter pathology. These findings demonstrate that even regions far remote from an infarct are affected predictably based on anatomical connectivity, and that white matter inflammation is an integral part of remote pathology. The delayed nature of this pathology makes it a valid target for preventative treatment, potentially with an extended time window of opportunity for therapeutic intervention using anti-inflammatory agents.

## Introduction

Stroke is a leading cause of disability in developed countries (Adamson et al., 2004; Mendis, 2013), with functional deficits arising both from neuronal death within the infarct core as well as from delayed secondary damage to neighboring or remote structures. While acute neuroprotection to prevent acute secondary damage within the peri-infarct region has been a focus of intense research efforts (Heiss, 2012; Majid, 2014), relatively few studies address the fact that delayed secondary damage often occurs to remote brain regions that are anatomically connected to the primary infarct by axonal projections. Although, the functional significance of affected remote brain regions is generally difficult to establish, some clinical studies suggest a link between remote damage and symptomatology (Demeurisse et al., 1990; Inagaki et al., 1992; Thiel et al., 2010), a phenomenon termed “diaschisis” (Finger et al., 2004). Select clinical reports also demonstrate that remote changes post-stroke are readily measurable by imaging modalities such as computed tomography (CT; Tamura et al., 1991), magnetic resonance imaging (MRI; Nakane et al., 2002; Duering et al., 2012; Yassi et al., 2015), and positron emission tomography (PET; Pappata et al., 2000; Jacobs et al., 2012), and are in some cases detectable even at chronic stages, suggesting ongoing or prolonged pathology (Tamura et al., 1991; Thiel et al., 2010). Supporting clinical findings, studies in rats using middle cerebral artery occlusion (MCAO) as an ischemia model consistently show that distinct regions of the thalamus with axonal connections to the primary infarct develop gliosis, inflammation, and significant neuronal loss at delayed post-stroke time points (Fujie et al., 1990; Iizuka et al., 1990; Dihné et al., 2002; Loos et al., 2003). Experimental striatal lesions produce comparable pathology in distinct structures of the substantia nigra (Block et al., 2004, 2005), a phenomenon that translates to the clinic (Nakane et al., 1992). A range of pathophysiological mechanisms has been suggested to underlie these remote changes (Dihné et al., 2002; Block et al., 2005), and mechanisms may vary depending on the structures affected and the nature of their axonal connectivity. For example, Dihné et al. (2002) suggest disinhibition due to loss of GABAergic input to the thalamus as a potential pathophysiological mechanisms for thalamic degeneration in their MCAO model.

A commonality among all observations of remote neuronal damage is the axonal connectedness of these regions with the primary infarct site, suggesting a role for axonal degeneration and potential white matter pathology, which has received little experimental attention so far (Irving et al., 2001; Wakita et al., 2002; Moxon-Emre and Schlichter, 2010). Yet, recent clinical imaging studies report white matter inflammation in major fiber pathways at different time points after stroke, including several months into the recovery period (Uchino et al., 2004; Gerhard et al., 2005; Radlinska et al., 2009; Thiel et al., 2010). This delayed and in some cases persistent white matter pathology in the aftermath of a stroke has been suggested to be of functional relevance for post-stroke recovery (Thiel et al., 2010).

The delayed nature of secondary remote changes after stroke both in gray and white matter may widen the time window for therapeutic intervention beyond the acute stage. A more complete understanding of remote secondary damage will inform strategies aimed at preventing delayed damage processes, including white matter inflammation, which may be of significance in post-stroke recovery.

In the present study, we use a model of prefrontal cortex (PFC) stroke to characterize sites of far-remote secondary damage. This model contrasts the commonly used MCAO model, where the secondarily affected thalamus is still in relatively close proximity to the infarct. We set out to test two hypotheses: First, remote damage is limited to areas connected to the infarct core, and includes far-distant gray matter regions. Second, axonal projections connecting the infarct to remote brain regions degenerate, leading to white matter pathology.

## Methods

### Animals and surgical procedures

All procedures involving live animals were conducted in accordance with the Canadian Council for Animal Care guidelines and approved by the University of Prince Edward Island Animal Care Committee. Behavioral experiments conducted with these animals have been reported previously (Déziel et al., 2015). Briefly, rats underwent a set shifting test using a plus maze with brightness and texture cues, where they learned to associate a cue with a food reward. The cued feature (brightness or texture) was then changed in the set shift test. Rats were food deprived for 20 h prior to training sessions during 2 weeks before the stroke surgery and prior to testing on post-surgery days 8 and 9. On days 7, 14, 21, and 28 post-surgery, all animals were tested in a temporal object recognition task over the course of 240 min each day.

Thirteen male Sprague-Dawley rats aged 3.5–4 months and weighing 400–450 g were randomly assigned to either the stroke group (*n* = 7) or control (*n* = 6). Anesthesia was maintained with a 2–3% isoflurane mixture (PPC, Richmond Hill, Canada), and Xylocaine (AstraZeneca, Mississauga, Canada) was topically applied to the surgical site. Artificial cerebrospinal fluid (aCSF; composition: NaCl, 124 mM; KCL, 4 mM; NaH_2_PO_4_, 1.24 mM; MgSO_4_, 1.3 mM; CaCl_2_, 2 mM; NaHCO_3_, 26 mM; Glucose, 10 mM; control group) or aCSF containing 400 pmol endothelin-1 (ET-1, Calbiochem, Etobicoke, Canada; stroke group) were injected bilaterally (1 μL on each side) and targeted at specific coordinates relative to bregma as follows: anterior/posterior +3.0, medial/lateral ±0.7, dorsal/ventral −4.5, as described by Déziel et al. (2015, Figure 1A). The needles remained at the injection site for 4 min following injection and were then slowly retracted. The surgical site was then sutured, and Xylocaine was reapplied to the area. Each animal received a post-operative 2 mg/kg subcutaneous injection of butorphanol (Wyeth, Guelph, Canada) for analgesia.

**Figure 1:**
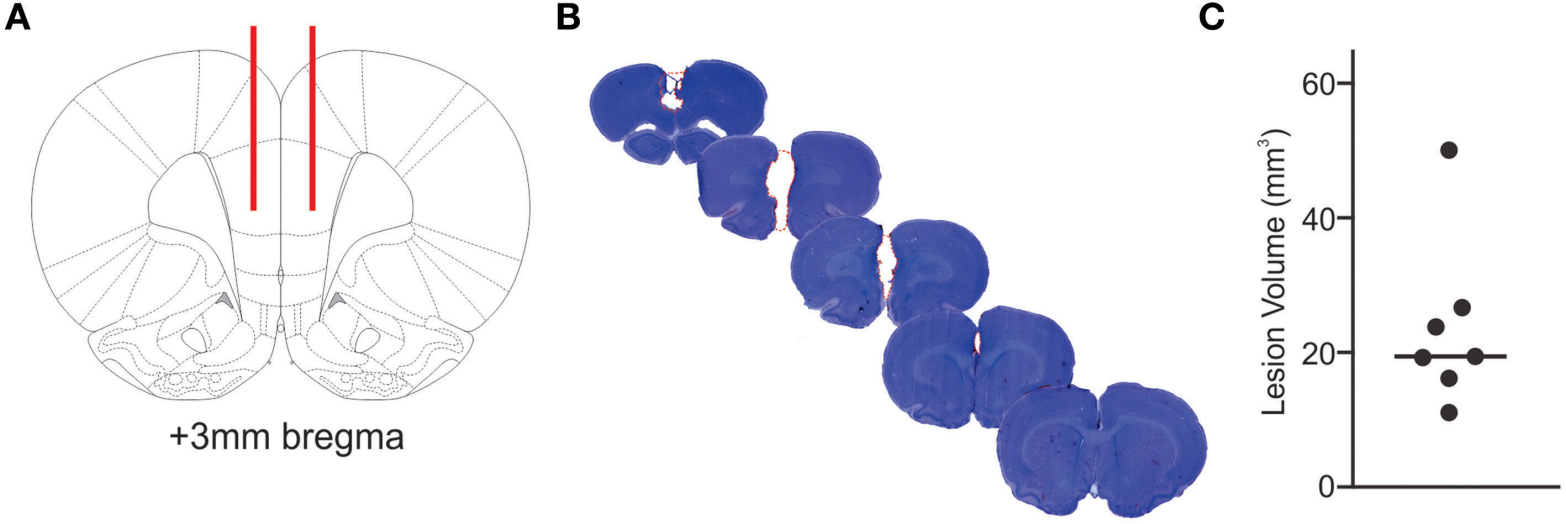
**Extent of infarcted tissue**. The locations for bilateral injection of ET-1 or saline into the prefrontal cortex are shown in red in a schematic cross section **(A)**. Tissue loss due to ET-1 injection is outlined by a red, dashed line in a series of cresyl violet stained coronal brain sections **(B)**, illustrating the extent of a representative ischemic lesion in the prefrontal cortex. The calculated volume of infarcted tissue for each ET-1 injected animal **(C)** demonstrates the variability of primary lesion sizes.

### Euthanasia and tissue processing

Rats were euthanized 28 days post-surgery with 4% isoflurane followed by decapitation. The brain was removed and post-fixed in 10% neutral buffered formalin for at least 48 h. The anterior portion of each brain spanning the infarcted region was sectioned at 100 μm thickness using a vibratome, and stained with cresyl violet as described by Déziel et al. (2015). The remaining posterior brain tissue was transferred to a cryoprotective 30% sucrose solution in phosphate-buffered saline (PBS). Free-floating 30 μm-thick tissue sections were collected in six series using a cryostat, and stored in cryoprotective solution (30% sucrose, 30% b/v ethylene glycol in 0.1M PB) at −20°C.

### Lesion analysis

Following cresyl violet staining, damaged tissue was identified by a lack of tissue integrity and abnormal cytological architecture. The area of damaged tissue was outlined in each section and measured using NIH ImageJ software. Each area measurement was then multiplied by the distance between sections (100 μm) to calculate the total lesion volume, as described in Déziel et al. (2015).

### Histochemistry

Brain sections were washed in 0.01 M phosphate buffered saline (PBS) for a total of 1 h (6 × 10 min), and then mounted on glass slides (Fisher Scientific Superfrost® Plus, Toronto, Canada) using 0.3% gelatin (Fisher Scientific, Toronto, Canada), and were dried overnight. Slides were incubated in 1% sodium hydroxide diluted in 80% ethanol for 5 min. They were then dehydrated in a graded series of ethanol (95% for 3 min, 70% for 3 min, and 50% for 2 min), washed in distilled water (3 × 1 min), and incubated in 0.06% potassium permanganate (Fisher Scientific) for 15 min on a shaker. The slides were washed with PBS (3 × 1 min) and placed in 0.0004% FluoroJade B (Chemicon, Etobicoke, Canada) for 20 min on shaker. Lastly, the slides were washed with PBS (3 × 1 min), dried in an incubator at 37°C for 25 min, cleared in xylene (Caledon Laboratories Ltd., Georgetown, Canada) for 1 min and coverslipped with Depex mounting medium (Electron Microscopy Sciences, Hatfield, USA).

### Immunohistochemistry

Immunohistochemistry was performed on free-floating sections washed in PBS for 1 h, then treated with 1% hydrogen peroxide in PBS on a shaker for 15 min to inhibit endogenous peroxidase activity. Slides to be immunolabeled with NeuN underwent antigen retrieval in 0.01 M citric acid solution at 95°C for 1 h.

Slides to be immunolabeled with NeuN, Iba-1, and OX-6 (antibody clone raised against MHC-II antigen) were then blocked with horse serum (Sigma-Aldrich, Oakville, Canada) in PBS-Triton X-100 (PBST) at a concentration of 1:50. Slides to be immunolabeled for myelin basic protein (MBP) were blocked with 10% goat serum in PBST. The primary antibodies used were mouse anti-NeuN (1:1000, Chemicon), rabbit anti-Iba-1 (1:1000, Wako Chemicals, Richmond, USA), and rabbit anti-MBP (1:200, ab40390, Abcam, Toronto, Canada), and mouse anti-OX-6 (1:1000, 554926, BD Pharmingen, Mississauga, Canada). The slides were incubated with primary antibody for 1 h on a shaker at room temperature, and then moved to a 4°C fridge overnight on a shaker. Following a PBS wash the next day, NeuN and OX-6 stained sections were incubated in horse anti-mouse IgG (1:500, Vector Laboratories Inc., Burlington, Canada) diluted in blocking buffer. Iba-1 stained sections were incubated in goat biotinylated anti-rabbit IgG (1:500, Vector Laboratories Inc.) in blocking buffer. MBP stained sections were incubated in goat biotinylated anti-rabbit IgG (1:500, Vector Laboratories Inc.) in blocking buffer.

Following incubation with secondary antibody, all sections were rinsed in PBS, then incubated in avidin-biotin complex (Vectastain® ABC Kit, Vector Laboratories Inc.) diluted in 0.01 M PBST for 1 h. They were then treated with 0.05% diaminobenzidine and 0.03% hydrogen peroxide in PBS for up to 1 min, until reaction products reached the intended color intensity. Sections were mounted with 0.3% gelatin on glass slides (Fisher Scientific), dried, then dehydrated with a graded series of ethanol (50, 70, 95, and 100%) for 5 min each, immersed in a solution of 50% ethanol and 50% xylene for 5 min, cleared in xylene for 10 minutes and coverslipped with Depex mounting medium.

### Histological analyses

Serial brain sections (at 180 μm intervals) ranging from about 2 mm posterior bregma to 4.7 mm posterior bregma were screened for FJB positive signal. Based on FJB positive signal, regions of interest were determined for all further analyses. Digital photomicrographs were taken in NIS Elements software (version 4.30.02, Nikon Instruments Inc., Melville, USA) using a fluorescent and light microscope (Nikon Eclipse Ni-U, Nikon Instruments Inc.). FJB signal intensity was rated using a graded scale from 0 to 4 in the dorsomedial nucleus of the thalamus (DMN), in the internal capsule (IC), and in the retrosplenial area (RSA) of the cortex (RSA). For each animal, 15 sections were used for FJB quantification and for determination of regions of interest for NeuN and Iba-1 analyses. Photomicrographs of NeuN, Iba-1, and MBP to be analyzed using ImageJ were converted into 8-bit images and threshold was established using ImageJ. NeuN positive cells were counted using the particle count function and Iba-1-positive as well as MBP positive signal was quantified by determining the percent area of signal in each selected ROI using ImageJ. Seven sections per animal were used for NeuN and Iba-1 analyses of the DMN and IC, and five sections per animal were used for the analysis of the RSA. All analyses in these ROIs were performed for the left and right hemisphere. OX-6 positive, MHC-II expressing cells were counted twice both in the left and right IC in three sections per animal. The average of both counts in each region was then used to calculate the sum of all six regions analyzed per animal. The percentage of MBP + signal area was measured in photomicrographs of the right and left IC in at least four sections per animal using ImageJ. Sample areas for all histological analyses were consistent across all animals for each region of interest. Histological results are expressed as the average value across all regions measured for each animal.

### Statistical analyses

Statistical comparisons were conducted using Graphpad Prism software (version 6 for Mac, GraphPad Software Inc., La Jolla, USA). FJB rating scores were compared using the Wilcoxon signed rank test. NeuN, Iba-1, OX-6, and MBP data sets were compared using Mann Whitney tests. Results are reported as mean ± standard error of the mean (SEM), and dot plots show the median.

## Results

### Extent of infarcted tissue

Bilateral ET-1 injection consistently resulted in ischemic tissue damage in areas of the PFC including the prelimbic cortex, medial orbital cortex, cingulate cortex, and premotor area (Figure 1B). ET-1 injection resulted in an average infarct volume of 23.78 ± 12.65 mm^3^, with the largest ischemic region measuring 50.08 mm^3^ and reaching from 4.7 to 0.5 mm anterior bregma (Figure 1C). Histology of the brains of control animals did not yield evidence of compromised tissue integrity within the injected region.

### Cellular degeneration in distinct remote brain regions

In order to identify potential regions of tissue damage remote from the infarct, brain sections posterior to the prefrontal cortical ischemic lesion as well as matched sections from control brains were screened for degenerating cells using FluoroJadeB (FJB) histochemistry. In all infarcted brains, the DMN of the thalamus, the RSA of the cortex and the IC took up FJB signal (Supplementary Figure 1). This analysis indicates that a prefrontal ischemic lesion results in well-defined regions of remote tissue damage, as close as 2.5 mm and as distant as 4.3 mm from the posterior border of the infarct. Based on these findings, the DMN, the RSA, and the IC were identified as regions of interest for further characterization of pathology.

### Histopathological findings in the thalamus

**Figure 2:**
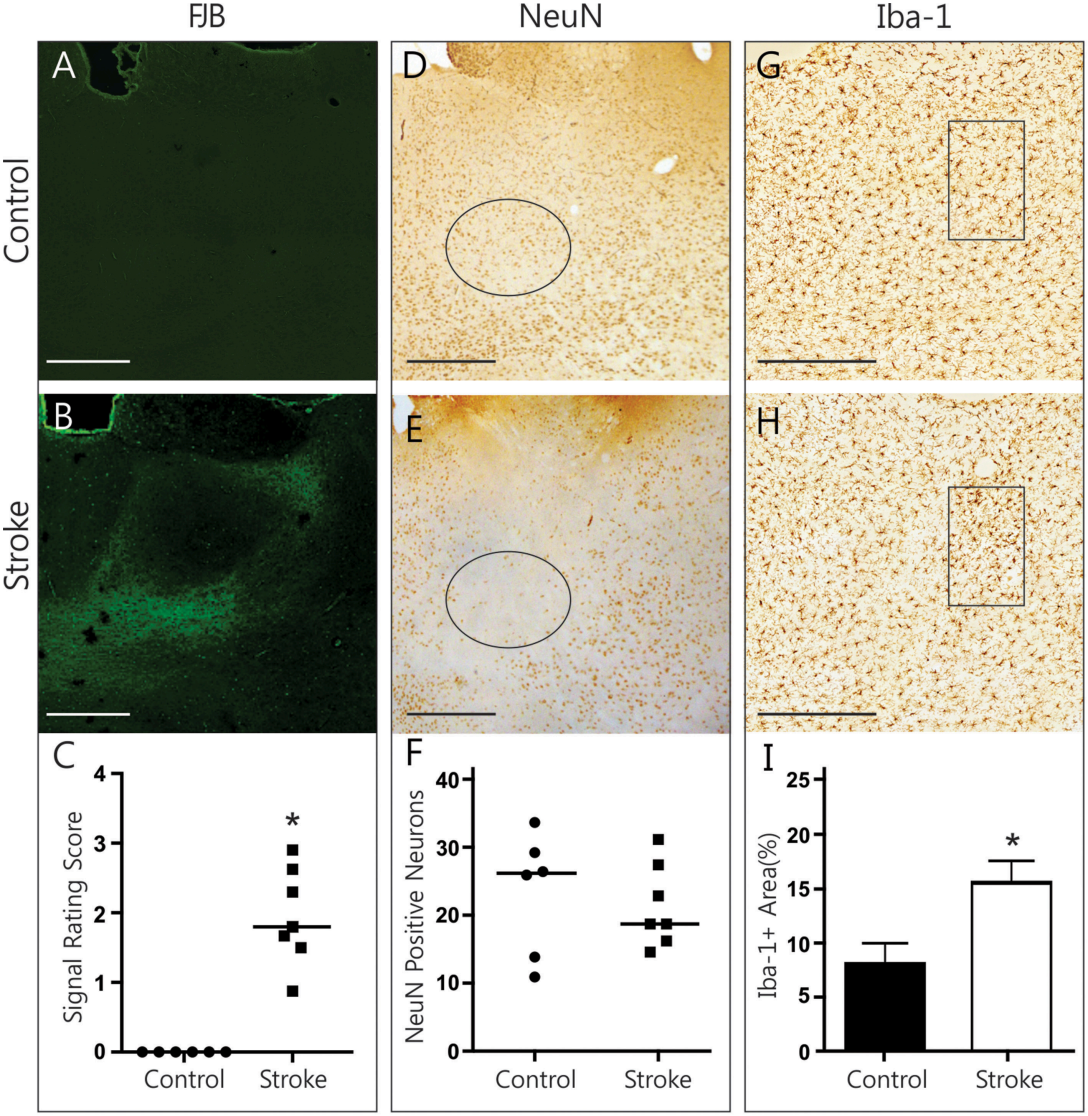
**Histopathological findings in the thalamus**. Representative photomicrographs show FJB signal **(A,B)**, NeuN immunolabeling **(D,E)**, and Iba-1 immunolabeling **(G,H)** in the DMN of control and ET-1 injected brains. Black circle **(D,E)** and rectangle **(G,H)** indicate sample areas used for quantification. Infarcted brains have significantly more FJB (**C**, Wilcoxon signed rank test, *p* = 0.016) and Iba-1-positive (**I**, Mann Whitney test, *p* = 0.022) signal in the DMN than control animals. No neuronal loss is evident in stroke brains **(F)**. Scale bars = 500 μm. ^*^p < 0.05.

FJB signal in the thalamus was clearly limited to the medial and lateral parts of the dorsomedial thalamic nucleus, sparing its central compartment (Figures 2A,B). Although, the DMN took up FJB signal bilaterally, no distinct cells were labeled. Rating of FJB signal indicated significantly higher FJB signal in the DMN (rating of 1.95 ± 0.26, *p* = 0.016, Figure 2C) of ET-1 injected animals, with control animals having a consistent score of “zero.” The diffuse, non-cellular FJB signal within the DMN does not indicate that cells were actively degenerating at this 28-day post-infarct time point. However, it is possible that degeneration may have already occurred and abated. To test whether neurodegeneration had taken place, NeuN-positive neurons were counted in the left and right lateral DMN (Figures 2D–F). Neuronal loss was not evident in this region in ET-1 injected animals (21.38 ± 2.30 cells) compared to control animals (23.33 ± 3.66 cells), as two control animals had comparatively low numbers of NeuN positive cells (Figure 2F). OX-6 positive cells were largely absent. To confirm that the positive FJB signal was not an artifact, we quantified Iba-1 positive microglia signal in the DMN (Figures 2G–I). Interestingly, the area of Iba-1 positive signal in the DMN was doubled in infarcted brains (15.58 ± 2.02% area) compared to control brains (8.08 ± 1.92% area, *p* = 0.022, Figure 2I). Such an increase in Iba-1 is a good indication of ongoing pathological processes, and may reflect both microglia recruitment and activation. This significant difference was measured to a similar extent in the left and the right DMN separately (not shown), indicating that both structures are involved in secondary damage processes or repair thereof at 28 days post-infarct.

### Histopathological findings in the cerebral cortex

**Figure 3:**
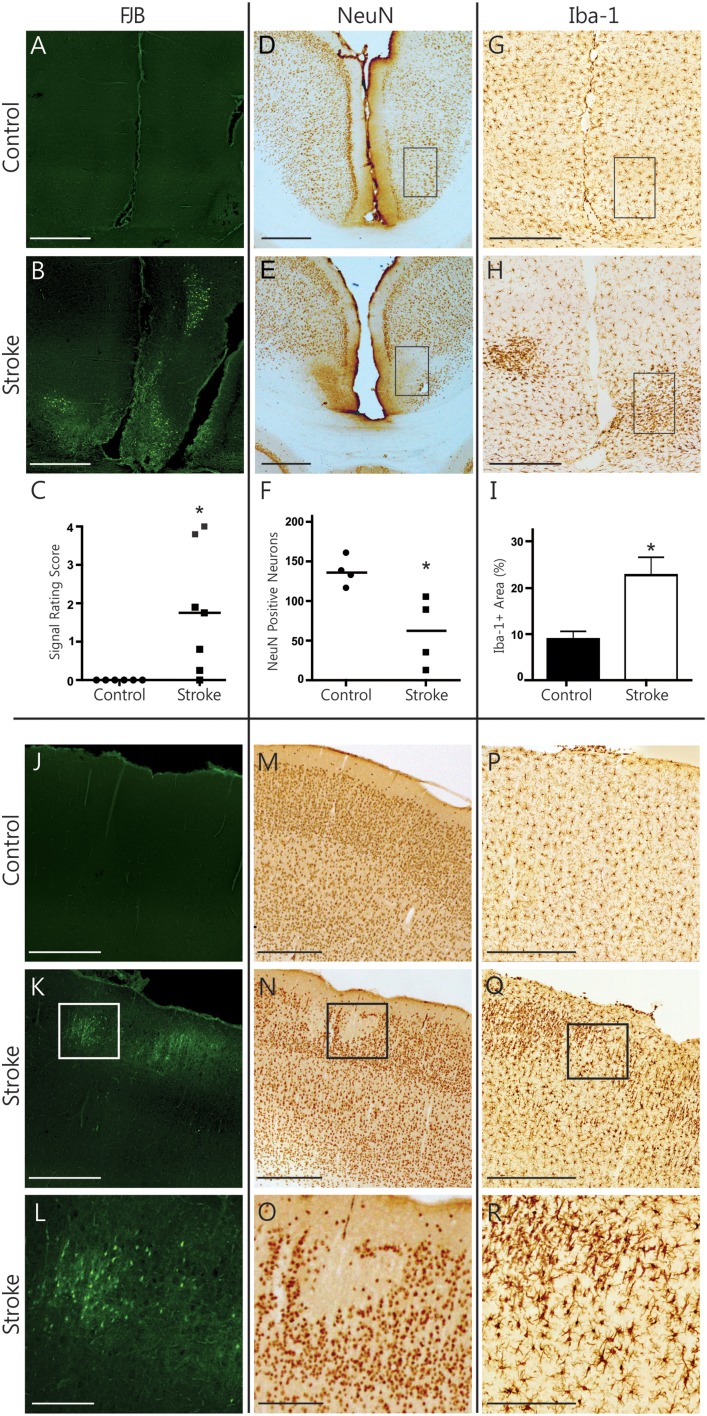
**Histopathological findings in the cerebral cortex**. Representative photomicrographs show FJB signal **(A,B)**, NeuN immunolabeling **(D,E)**, and Iba-1 immunolabeling **(G,H)** in the RSA of the cortex. Black rectangles **(D,E,G,H)** indicate sample areas used for quantification. Infarcted brains have significantly more FJB signal (**C**, Wilcoxon signed rank test, *p* = 0.031) and show significant neuronal loss (**F**, Mann Whitney, *p* = 0.029) in the RSA. Iba-1-positive signal is significantly higher (**I**, Mann Whitney test, *p* = 0.014) in infarcted brains. Photomicrographs of the SM1 area of the cortex from one stroke animal demonstrate remote cortical pathology based on positive FJB signal **(J–L)**, focal loss of NeuN signal **(M–O)** and accumulation of Iba-positive microglia **(P–R)**. Scale bars = 500 μm. The bottom row shows magnified images of the areas outlined in K, N and Q, respectively. Scale bars = 250 μm. ^*^p < 0.05.

In the RSA of the cortex, ongoing degeneration was captured by FJB labeled cells in distinct clusters (Figures 3A,B). The RSA of ET-1 injected animals had a significantly higher rating of FJB signal than controls (rating of 1.79 ± 0.61 vs. 0, *p* = 0.031, Figure 3C). Quantifying the number of NeuN positive neurons in the RSA revealed significant neuronal loss in infarcted brains (60.80 ± 21.91 cells vs. 137.4 ± 9.18 cells, *p* = 0.029, Figures 3D–F). In accordance with this, the area of Iba-1 positive microglia signal more than doubled in ET-1 injected (22.70 ± 3.86% area) compared to control animals (8.92 ± 1.69% area, *p* = 0.014, Figures 3G–I). Iba-1 signal in the RSA was also more lateralized in ET-1 injected (4.08 ± 0.94% area more on one side) than in control animals (0.61 ± 0.18% area difference between sides, *p* = 0.001, not shown). The brain with the largest stroke volume displayed a well-defined cluster of focal FJB signal in the SM1 region of the cortex (Figures 3J–L), which was accompanied by visible neuronal loss (Figures 3M–O) and microglia recruitment (Figures 3P–R). OX-6 positive, MHC-II expressing activated microglia were not found in any cortical region. Taken together, these results indicate that secondary damage processes are ongoing in cortical regions remote from and connected to the infarct, resulting in significant neuronal degeneration.

### Histopathological findings in the internal capsule

**Figure 4:**
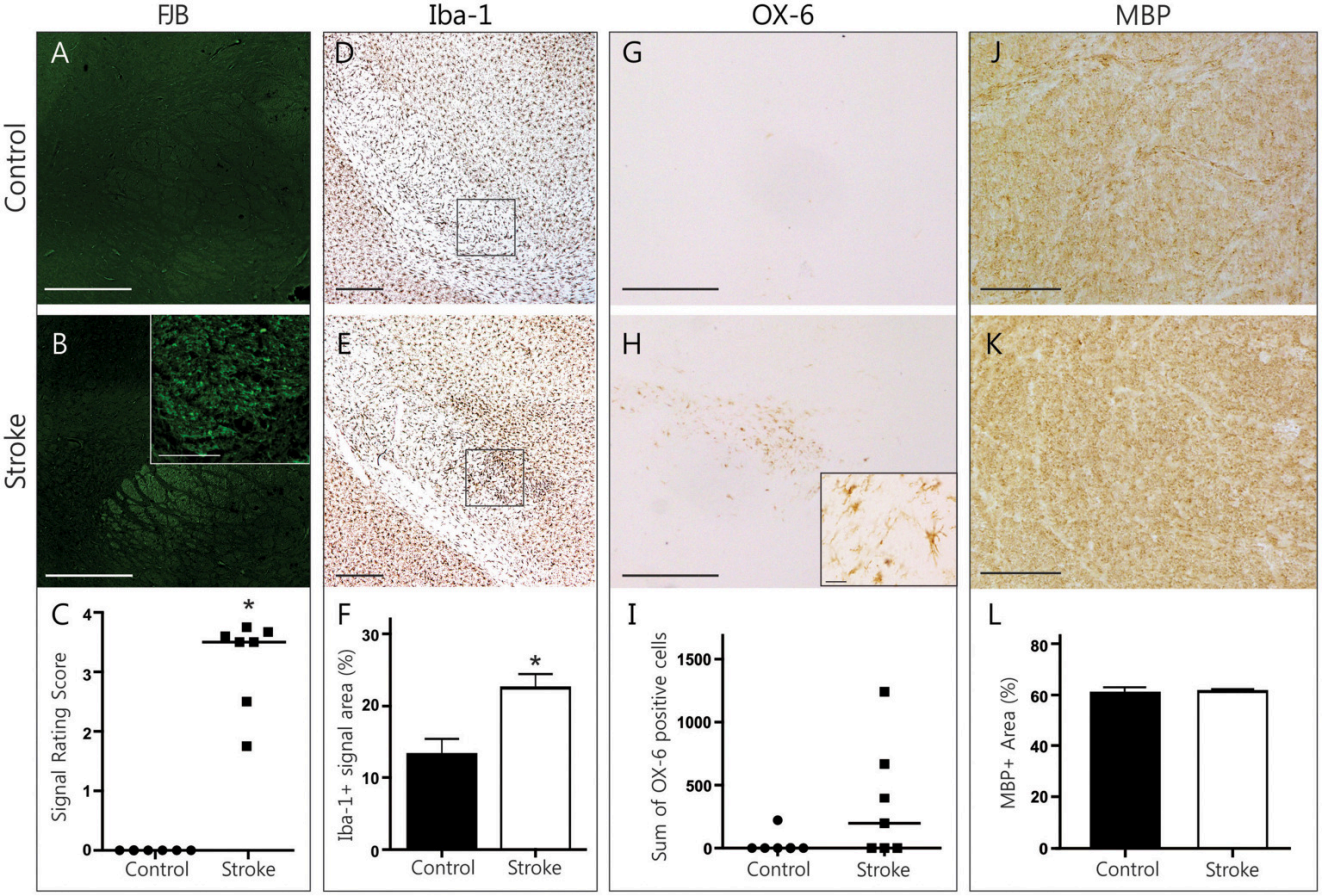
**Histopathological findings in the internal capsule**. Representative photomicrographs of FJB stained coronal brain sections from a control **(A)** and stroke **(B)** animal illustrate the distinct FJB signal in the internal capsule only found in stroke animals (**C**, Wilcoxon signed rank test, *p* = 0.016,). Scale bars = 500 μm, magnified inlet in B = 50 μm. Photomicrographs of Iba-1 positive microglia (**D,E**, scale bars = 500 μm) indicate sample areas (black rectangle) used for quantification. Infarcted brains had a significantly higher Iba-1+ signal area (**F**, Mann Whitney test, *p* = 0.035). The internal capsule of infarcted brains tended to have a higher number of OX-6 positive cells (**G,H**, scale bars = 500 μm, magnified inset in H = 25 μm) compared to controls (**I**, Mann Whitney test, *p* = 0.15). Photomicrographs of myelin basic protein signal (**J,K**, scale bars = 100 μm) represent sample areas used for quantification. MBP signal area was not different between groups **(L)**. ^*^p < 0.05.

FJB-positive cell bodies were scarce in the IC, as expected in white matter regions. Most FJB signal was in the shape of dots and short lines, which is consistent with the morphology of cross-sectioned axons (Figures 4A,B). Animals in the ET-1 group had significantly higher FJB signal ratings than controls (rating of 3.181 ± 0.29 vs. 0, *p* = 0.016, Figure 4C), suggesting the presence of degenerating axons that project mostly perpendicularly to the plane of section. The area of Iba-1 signal in this region was significantly higher in ET-1 injected (22.56 ± 1.95% area) compared to control brains (13.27 ± 2.18% area, *p* = 0.035, Figures 4D–F), with similar results in either hemisphere (not shown). In line with this, we found OX-6 positive, activated microglia bilaterally in the IC in 4 out of 7 animals in the ET-1 group (357.7 ± 175.2 cells vs. 37 ± 37 cells, *p* = 0.15, Figures 4G–I). To find out whether axonal degeneration and accompanying inflammation may have resulted in a measurable degree of demyelination, we quantified MBP signal within the left and right IC (Figures 4J–L). There was no indication of myelin loss when ET-1 injected brains were compared to control brains (Figure 4L). Taken together, this evidence points toward white matter inflammation and degeneration in axonal pathways that connect distant brain regions with one another.

## Discussion

Remote secondary damage after stroke in the rat has been primarily studied using the MCAO model (Fujie et al., 1990; Iizuka et al., 1990; Dihné et al., 2002). While this model offers the opportunity of studying the break-down of well-defined connections within the thalamocortical circuits, the thalamic areas that suffer secondary pathology (i.e., ventroposterior and reticular nuclei of the thalamus Dihné et al., 2002), are still in relatively close proximity to the infarct core. Since MCAO lesions are rarely restricted to the cortex, but often include subcortical structures and produce widespread edema, primary, and secondary damage are occurring in spatial proximity to and acutely after the primary infarct. The herein described PFC stroke model in the rat offers several advantages for the study of remote degeneration. First, the stroke is limited to the anterior part of the rat's brain, therefore secondary damage can be studied in remote areas posterior to the infarct border and in clear separation from the infarct core and from potential secondary expansion of the core into peri-infarct regions. Second, the considerable spatial distance between primary infarct and remote damage may offer an extended time window to study secondary damage processes, which future studies addressing the temporal development of these lesions will clarify. This may be an asset for future investigations into mechanisms and treatment options. Third, the predictable involvement of the IC in remote pathology presents a unique opportunity to further study the pathophysiological role of white matter inflammation after stroke. Post-stroke white matter inflammation has gained attention in the clinical community, both due to its link to prognosis and recovery and due to its persistence months after a stroke incident (Gerhard et al., 2005; Thiel et al., 2010). White matter inflammation is not only an interesting finding regarding the pathophysiology of secondary damage to regions connected to the infarct core, but also of relevance for potential secondary damage to intact axons projecting in close proximity to those undergoing Wallerian degeneration. It has been demonstrated in the past that inflammation, which is considered a hallmark of Wallerian degeneration (Vargas and Barres, 2007; Wang et al., 2009), can lead to secondary damage within degenerating white matter tracts (Weishaupt et al., 2010). However, it needs to be emphasized that a finding of microglia recruitment/activation by itself, although a sign of pathology, does not necessarily imply a harmful function of inflammation. This caution applies to the microglia recruitment/activation observed here in the IC as well as in the thalamus. In recent years, the body of literature characterizing a spectrum of microglia phenotypes and their polarization into classically activated M1 (generally considered pro-inflammatory and potentially harmful) and alternatively activated M2 (considered anti-inflammatory and beneficial for repair and plasticity) microglia has grown immensely (Kigerl et al., 2009; Perego et al., 2011; Hu et al., 2012). This distinction has shown that certain alternatively activated microglia can be beneficial for healing processes (Cherry et al., 2014). In the present study, we found activated microglia cells expressing MHC-II (OX-6 positive cells), a marker predominantly seen in M1 polarization, in the IC of four out of seven stroke brains. The fact that these cells were not present in the DMN or the RSA may point to a different time scale and/or a different kind of inflammation in remote white matter vs. gray matter damage after stroke. This is not surprising given that even in the healthy brain, resident microglia phenotypes vary across brain regions (Lawson et al., 1990). It would be interesting to further elucidate the nature of the inflammatory processes found in remote brain regions after stroke in future studies, as modifying this inflammation may evolve to be a worthwhile therapeutic strategy for stroke survivors.

Significant microglia recruitment/activation at sites connected to the PFC infarct is a consistent finding in the present study, which is accompanied by neuronal loss and ongoing neuronal degeneration in the RSA of the cortex. Although, not accompanied by measurable neuronal loss in the thalamus, the fact that inflammation and signs of degeneration are present at 28 days post-stroke indicates either a delayed, evolving pathology, or, alternatively, a lingering pathology that developed earlier after the infarct. A number of studies addressing secondary damage in rats study recovery times limited to 14 days (Dihné et al., 2002; Loos et al., 2003; Moxon-Emre and Schlichter, 2010), with notable exceptions (Fujie et al., 1990; Iizuka et al., 1990). Our results are in accordance with the latter reports (Fujie et al., 1990; Iizuka et al., 1990), suggesting that far-remote pathology exists at more chronic post-stroke time points. This finding is also in line with imaging studies in patients that report signs of atrophy and inflammation remote from an infarct several months after the incident (Gerhard et al., 2005; Thiel et al., 2010; Duering et al., 2012). More work is needed to answer the question what exact time course far-remote pathologies follow.

While the present study is restricted to the observation of pathology at one moment in time only, it does support the hypothesis that remote brain regions directly connected to the infarct are actively affected at this chronic time point. We verified this conclusion by comparing those regions we found to be FJB-positive (after scanning through all brain sections) to the Allen Mouse Brain Connectivity Atlas (Oh et al., 2014), which elegantly visualizes the anatomical connectivity of prefrontal cortical areas with other brain structures. Using this resource, connections from the prelimbic cortex, medial orbital cortex, and cingulate cortex, structures that suffered ischemia in our model, can be traced to the IC, the RSA of the cortex, as well as the medial and lateral parts of the DMN, sparing the central part of the DMN (Supplementary Figure 1). This very distinct reciprocal connectivity between the DMN and the PFC has been reported in the past, for example by injection of anterograde and retrograde tracers into individual substructures of the DMN and the PFC (Groenewegen, 1988).

In conclusion, PFC stroke in the rat produces a predictable and well-defined pattern of remote and delayed secondary damage exclusively in brain regions connected to the PFC. Future studies are necessary to address the limitations of the present study, such as the nature of inflammation, the time course of remote secondary pathology, and the mechanistic details underlying this pathology.

## Author contributions

NW designed the histology experiment, conducted some of the histology and data analysis and wrote the manuscript. AZ conducted most of the histology, performed data analysis and contributed to the manuscript. RD and RT conceived and performed the animal experiment and improved drafts of the manuscript. SW contributed to designing the experiment, to data analysis and writing the manuscript.

## Funding

We acknowledge funding support from the Ontario Mental Health Foundation (OMHF Fellowship to NW), the National Sciences and Engineering Research Council of Canada (NSERC: 418489), Canadian Foundation for Innovation (CFI: 34213), Canadian Consortium for Neurodegeneration in Aging (CCNA), and Canadian Institutes of Health Research (CIHR: 126127) grants to SW, NSERC, and Innovation PEI (scholarships to RD) and Atlantic Innovation Fund (AIF grant 193639 to RT).

### Conflict of interest statement

The authors declare that the research was conducted in the absence of any commercial or financial relationships that could be construed as a potential conflict of interest.

## Abbreviations

DMN: dorsomedial nucleus of thalamus
ET-1: Endothelin-1
FJB: FluoroJade B
IC: Internal capsule
MBP: myelin basic protein
MCAO: middle cerebral artery occlusion
PBS: phosphate-buffered saline
PFC: prefrontal cortex
RSA: retrosplenial area of cerebral cortex.
