# Supplementary figures and images for: Prefrontal Ischemia in the Rat Leads to Secondary Damage and Inflammation in Remote Gray and White Matter Regions
Source: Front Neurosci. 2016 Mar 2;10:81. doi: 10.3389/fnins.2016.00081 (PMC4773446; doi:10.3389/fnins.2016.00081)

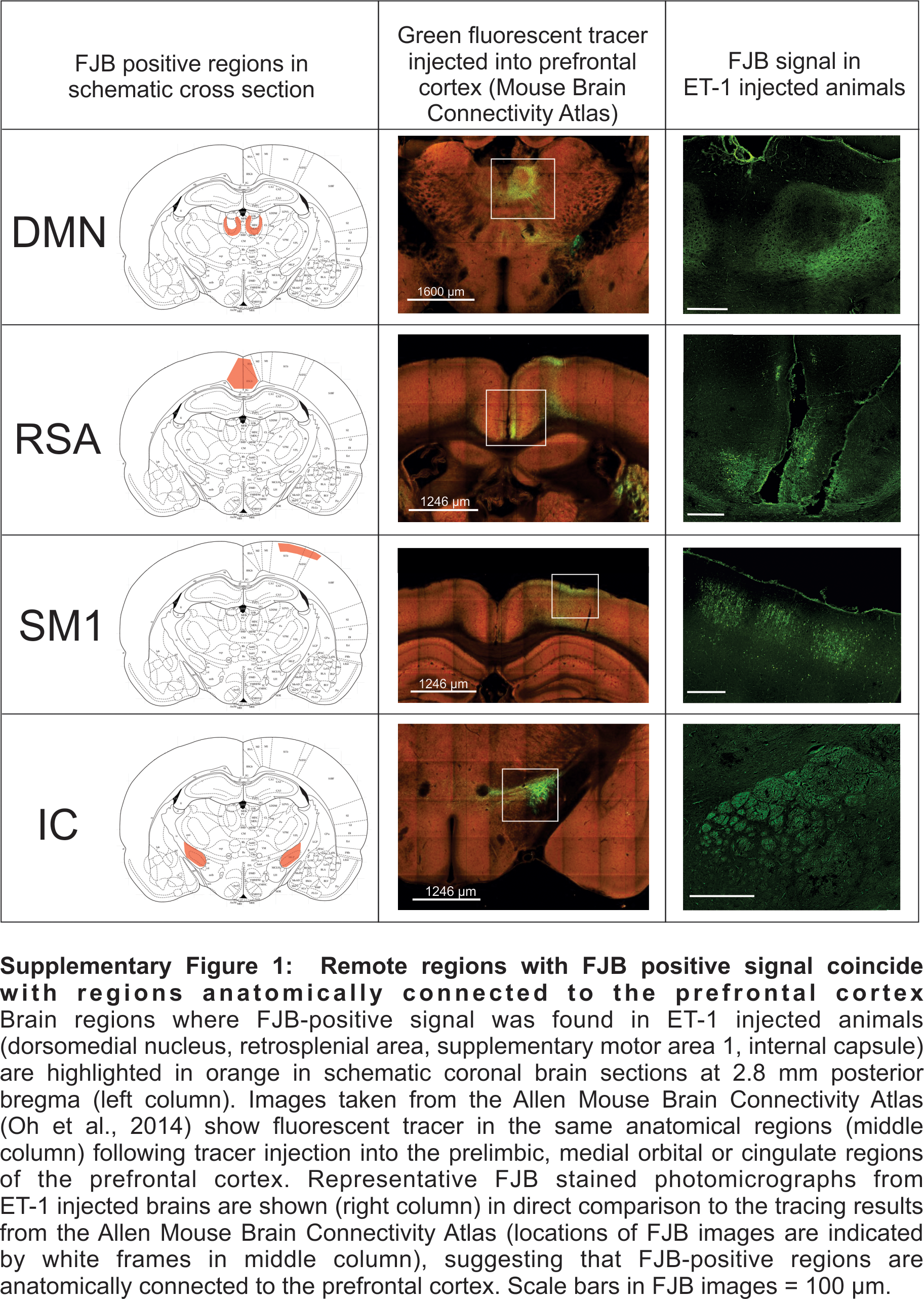

Supplement: Supplementary file 1 [file Image1.TIF]
